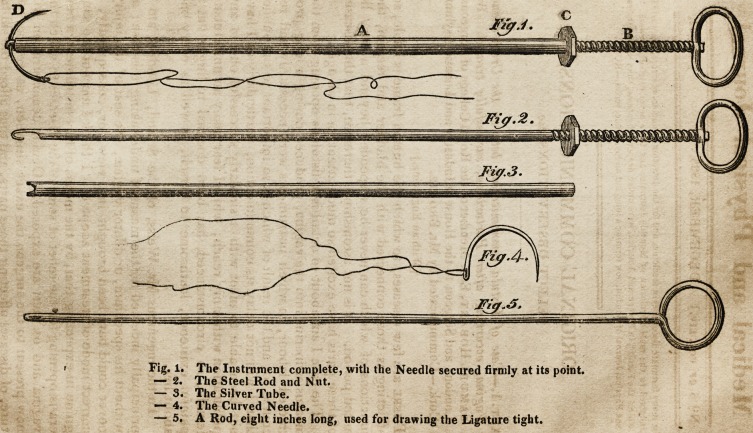# Case of Fistula Communicating between the Urethra and Vagina, Occurring after Parturition, Successfully Treated

**Published:** 1825-12

**Authors:** Samuel Hobart

**Affiliations:** Member of the Royal College of Surgeons, London; and Surgeon to the House of Industry and Lunatic Asylum, Cork.


Fig. 1. The Instrnment complete, with the Needle secured firmly at its point.

— 2. The Steel Rod and Nut.

— 3. The Silver Tube.

— 4. The Curved Needle.

— 5. A Rod, eight inches long, used for drawing the Ligature tight.

THE LONDON

### Medical and Physical Journal.

Nº 6 OF VOL. LIV.] DECEMBER, 1825. [No. 322.

For many fortunate discoveries in medicine, and for the detection of numerous errors, the world is indebted to the rapid circulation of Monthly Journals; and there never existed any work, to which the Faculty, in Europe and America, were under deeper obligations, than to the Medical and Physical Journal of London, now forming a long, but an invaluable, series.—RUSH.

### ORIGINAL COMMUNICATIONS,

### SELECT OBSERVATIONS, &c.

ART. I.—*Case of Fistula communicating between the Urethra and Vagina, occurring after Parturition, successfully treated.* By SAMUEL HOBART, Esq. Member of the Royal College of Surgeons, London; and Surgeon to the House of Industry and Lunatic Asylum, Cork.—Communicated by Mr. BRODIE.

[With an Engraving.]

THERE are few diseases, to which the human frame is liable, so afflicting as those connected with the bladder and rectum, the functions of which can never be suspended beyond a *very* limited period. It is not astonishing, therefore, that the diseases of these parts have excited so much attention in all ages.

The case I am about to detail is a fistulous opening from the meatus urinarius into the vagina, produced during a long protracted labour, in which instruments were necessarily had recourse to; and the result of which is so important to the public, that I will not delay its communication, particularly as I have not been able to meet with a similarly successful one in any publication within my reach; while I fear many an unfortunate female, so circumstanced, has been doomed to linger out a painful existence, loathsome to herself, as well as every one about her.

To the ingenious Mr. Weiss, of the Strand, London, am I principally indebted for the means of undertaking this case; as without his speculum, or dilator vaginæ, the seat of the injury never could have been brought into view, or, consequently, a correct mode of treatment adopted. I think I am not too sanguine in expecting that, by the means of this instrument, a new era is opening for the relief of some of the diseases to which the vagina and os uteri are exposed. Ocular demonstration will now present to the surgeon the exact situation, as well as nature, of diseases affecting those parts, and thus afford the skilful practitioner an opportunity of applying and changing his remedies according to *appearances*, and not, as heretofore, according to the imaginary progress of the case. The instruments used in the present instance are few and simple ; and even the needle may be changed in its situation, so as to alter its curve, without removing it from the vagina, merely by loosening the nut marked C, pushing the rod B a little down, and, with a long forceps, so changing its position, as to include in the ligature as much or as little as may be necessary. This is an object of great importance, as it at once relieves the operator from any difficulty which the first plunge, or rather incision, may induce. I shall now proceed to the detail of the case.

Catherine Mahony, a healthy country woman, aged twenty-six, applied to me, July 17, 1825, for relief from the following distressing symptoms : inability to retain her urine for one instant, and consequent excoriation and ulceration of the nates and thighs, arising from a considerable opening, by which a communication was formed between the meatus urinarius and the vagina. On dilating the vagina by means of Weiss's instrument, I with some difficulty (in consequence of the anterior portion of the vagina being considerably thickened,) succeeded in obtaining a satisfactory view of the opening, which was nearly an oval, its longer diameter being in the direction of the vagina, situated about two and a half inches from the os externum, and sufficiently large to admit the point of the finger ; its edges were quite smooth and callous. She gave the following history of her case :—About ten months previously she was, after a tedious labour of five days, delivered by means of the perforator and crotchet; and from that period the water continued to flow from the vagina, none ever passing through the orifice of the urethra.

The plan recommended by Desault of introducing a pessary into the vagina, and keeping a catheter in the bladder, was adopted, and persisted in for two months, without the slightest improvement. The edges were likewise touched with caustic, which was ineffectual, because the parts were not kept in actual contact for a sufficient length of time to cause their union. The case was then looked on as utterly hopeless, and she remained in the wretched situation above stated until I saw her. The actual cautery had been applied a few weeks before she came under my care, but was not productive of any advantage.

It appeared to me that, if I could keep a catheter constantly in the bladder, and bring the edges of the opening together by means of the bloody suture, after having destroyed the surface with caustic, they would unite. It was exceedingly difficult, in consequence of the depth of the opening in the vagina, to apply the suture: for this purpose I contrived an instrument for passing the needle through the lips of the wound, which answered my utmost expectations. This instrument (as represented in Fig. 1,) consists of four parts: a silver tube, or canula, A, about seven inches in length, and of the bore of a small goose-quill; a steel rod, B, passing through the tube freely, but fitting the bore accurately, about two inches longer than the tube, and terminating in a ring at the top, and a hook of peculiar form at the point, as more fully described in the accompanying drawing. On the rod, for about two and a half inches from the ring, is cut a screw, for the nut C to traverse upon. This steel rod, B, is introduced through the silver tube, A, until the hook at the end appears; which hook seizes the curved needle, D, and, by drawing up the rod, the needle is held tight across the end of the tube, where two notches are made (as in Fig. 3,) for its reception, and where it becomes firmly fixed in any position the operator chooses to place it, by screwing forward the nut, C, on the rod, so as to press upon the needle at the end of the tube.

The mode of using this instrument will, I think, be easily understood. The patient being properly placed on her knees, with her head as much below them as she can bear, and the parts to be united by the ligature being previously brought to a proper state; the vagina is then dilated by Weiss's speculum, which is held by an assistant, whilst the operator introduces the instrument represented in Fig. 1, (with the convex surface of the needle to the sacrum,) through the posterior edge of the wound. He then loosens its hold, by unscrewing one or two turns of the nut, C; and, having with a long forceps drawn the needle through, re-adapts it, and proceeds to pass it through the near or pubal edge of the opening. The needle having been brought through as before, it is again to be fastened at the end of the rod, so as to enable the operator, with the aid of the instrument marked No. 5, to fasten the ligature over the wound, in the same manner as uterine or nasal polypi are secured.

With regard to retaining the catheter constantly in the bladder, I accomplished that very material point in the following manner:—I procured an oval shield of wood, about three inches in diameter, in the centre of which I made a small hole, in which was fixed firmly the open end of the catheter; and, by means of a single tape fastened to this board below, and a double one above, I secured the catheter in its place, by passing the single tape between the nates, and fastening it and the two others to a band, which was passed round the waist for that purpose.

I found it necessary to apply the lunar caustic several times, as the sloughs were thrown off irregularly, and as it is absolutely necessary that the surface should be completely granulating all round the edges before the parts are brought into apposition. When the edges of the wound were in a fit state, I applied two ligatures made of strong silk, saturated in melted wax, to prevent the incrustation of calcareous matter, the disadvantages of which are obvious. The ligatures were allowed to remain in fourteen days, and were then removed with the greatest facility; the wax having completely prevented any earthy deposits. In three days after, the catheter was removed; when the urine flowed through the urethra, without escaping into the vagina.

In consequence of the necessity of wearing the catheter so long, and thus rendering the sphincter of the bladder in some measure passive, it did not recover its powers for several days. She is now able to retain her urine for many hours; and I have no doubt that, by the aid of cold bathing and other tonics, the full and healthy action of this viscus will be completely restored.

I am indebted to Drs. Woodroffe, Pitcairn, Bull, and Townsend, for their assistance and advice during the operation and progress of the case.

*Cork; August* 31, 1825.

## Figures and Tables

**Fig. 1. Fig. 2. Fig. 3. Fig. 4. Fig. 5. f1:**