# Errata

**Published:** 1826-01

**Authors:** 


					In the December Number, p. 450, Jor mineral read miueral waters.
453, for Blombieres read Plombieres.
454, for Selerite read Selenite.
455, for Castlehead read Castleleod.

				

